# Neurobrucellosis with negative serological examination: a case report and literature review

**DOI:** 10.3389/fmed.2025.1583891

**Published:** 2025-06-06

**Authors:** Boyao Yuan, Taotao Jiang, Jingjing Han, Manxia Wang

**Affiliations:** Department of Neurology, Lanzhou University Second Hospital, Lanzhou, China

**Keywords:** neurobrucellosis, metagenomic next-generation sequencing, diagnosis, treatment, Brucella

## Abstract

Neurobrucellosis is an uncommon occurrence that can arise as a consequence of brucellosis. However, its clinical symptoms are severe and have the potential to be life-threatening. Timely detection, prompt diagnosis, and early treatment are crucial factors. Clinically, the gold standard for diagnosing pathogenic microorganisms is through culture. However, this method is hindered by its lengthy culture duration, low rate of positive results, and the absence of typical clinical signs of neurobrucellosis. Consequently, misdiagnosis and delayed treatment are common. Metagenomics next-generation sequencing (mNGS) technology is a novel approach in microbiological diagnosis that enables the simultaneous detection of all microorganisms present in a sample, including viruses, bacteria, fungus, and parasites. This method holds significant diagnostic significance for viral disorders affecting the central nervous system. This paper reports a case of neurobrucellosis detected by mNGS after a negative serological test, as well as a review of the relevant literature.

## Introduction

Neurobrucellosis is a special type of brucellosis, which mainly refers to the disease caused by brucellosis invading the central nervous system (CNS) (1). Neurobrucellosis is relatively rare, but its clinical manifestations are serious, accounting for about 2–10% of all cases (2). Patients with neurobrucellosis may show persistent headache, stiff neck, disturbance of consciousness, seizure, muscle weakness, paresthesia and even paralysis (3, 4). In some serious cases, neurobrucellosis may lead to serious complications such as meningitis (3, 5), brain abscess (3), cerebral infarction (6), and even life-threatening. However, in a clinical setting, the disease’s start, evolution, and prognosis are complex and lack traditional clinical signs, making the diagnosis very challenging. While the culture of pathogenic microorganisms is considered the most reliable method for diagnosis, its lengthy culture period and low rate of positive results make clinical diagnosis primarily reliant on serological examination and associated neurological symptoms. These include tests such as standard Tube agglutination, anti-human globulin, Tiger red plate agglutination, and polymerase chain reaction technology (7). Nevertheless, numerous practitioners dismiss the diagnosis following a negative serological auxiliary screening, resulting in patients overlooking the optimal therapy opportunity and experiencing significant neurological impairment. Hence, it is crucial to identify a detection technique that can accurately and promptly diagnose diseases during their first stages.

Metagenome sequencing primarily utilizes high-throughput sequencing technologies, such as next-generation sequencing or second-generation sequencing, to analyze clinical samples using metagenomics. This allows for the extraction of various biological information, including species classification, serotype, drug resistance, and virulence of pathogens (8). It possesses the attributes of rapid detection speed, affordable cost, extensive coverage, exceptional precision, and high output (9). Additionally, it has the capability to conduct hundreds of thousands or even millions of sequences simultaneously. By evaluating the computer-obtained sequencing data, researchers can obtain entire DNA sequence information (9), which is currently a prominent area of research. This research presents a case of neurobrucellosis in which the serological screening for Brucella was negative, but mNGS was positive. As a result, the patient received timely and suitable antibiotic treatment, leading to satisfactory clinical outcomes.

## Case description

### General information

**Figure 1:**
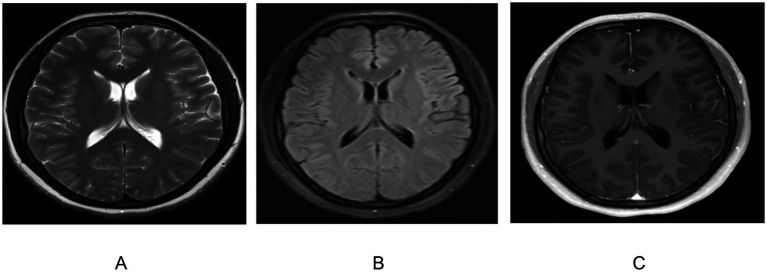
Brain magnetic resonance imaging (MRI) was performed after admission: **(A)** T2WI; **(B)** T2FLAIR; **(C)** Enhanced MRI.

A 35-year-old female patient was admitted to the Department of Neurology at the Second Hospital of Lanzhou University due to experiencing a persistent headache for over 20 days. Before admission, the patient had a headache after a cold, both sides of the top of the spleen, swelling pain, lasting for several hours can be relieved. Accompanied by nausea, non-spraying vomiting, fearless fever, consulted the local clinics and hospitals, given glycerol therapy such as hypothermia, However, the treatment had limited effectiveness. There were no obvious abnormalities in physical examination and specialist examination. Auxiliary examination after admission: routine blood examination: White blood cell 3.18 × 10^9^/L, and other normal results. Cerebrospinal fluid (CSF) examination: the pressure is 260mmH_2_O, the White blood cells are 597.00 × 10^6^/L, the CSF protein is 1.22 g/L, the glucose is less than 1.0 mmol/L, the chlorine is 118.0 mmol/L, and the infection of bacteria and fungi in CSF is negative. Biochemical examination: total cholesterol 5.63 mmol/L, low density lipoprotein 3.62 mmol/L, apolipoprotein 1.83 g/L and homocysteine 17.4 *μ* mol/L. Electrocardiogram, cardiac color Doppler ultrasound, abdominal color Doppler ultrasound, abdominal CT and head magnetic resonance imaging also showed no obvious abnormalities (Figure 1).

### mNGS detection and bioinformatics analysis

The mNGS procedures followed standard aseptic technique. First, nucleic acids were extracted from 0.4 mL CSF using the DNA/RNA Extraction Kit (GENSTONE BIOTECH, China) according to the instructions. Thereafter, the DNA library was constructed by using the Metagenomic DNA Detection Kit for Pathogenic Microorganisms (KingCreate Biotechnology, China) according to its manual. Finally, qualified libraries with SE75 reads length were sequenced on a NextSeq 550 platform (Illumina, CA, United States). To ensure the subsequent assembly and analysis results were microbial sequences, Filter 0.22.0 was used to perform basic quality control on sequencing, and remove adapters, inferior-quality reads (with average quality score < 20), N-terminal sequence (with single sequence with N bases > 5), short-sequence (length < 300 bp), low complexity and repeated sequences. Bwa 0.7.17, bowtie 2.4.2 and BLAST 2.11.0 were applied to compare the quality control data with NCBI nt database. Auto_anno 2.2 was used for microbial species annotation.

### Diagnosis and treatment process

Based on the patient’s primary clinical symptoms, such as headache, as well as the findings of blood routine and CSF investigation, we have a suspicion that the patient might be suffering from an infectious condition of the nervous system, potentially tuberculous meningitis or encephalitis. Nevertheless, the prompt identification of tuberculosis nucleic acid and rifampicin resistance yielded negative results, as did the quantitative detection of tuberculosis DNA in cerebral fluid. Following that, we conducted a comprehensive and meticulous epidemiological investigation on the patient. The investigation results showed that there was no farming of livestock such as cattle and sheep in the patient’s home. It is worth noting that before being admitted to the hospital, due to suffering from neck pain, the patient had used the wool from a neighbor’s house for external application treatment. Based on this special exposure history, we highly suspect that the patient has neurobrucellosis. Nevertheless, the serological test for Brucella yielded negative results in both the Tiger red agglutination test and the Tube agglutination test. The cerebral fluid culture for fungus and bacteria is negative, although the persistent headache symptoms continue during the diagnostic stage. Afterwards, the pathogen in the patient’s CSF was identified by mNGS. The analysis revealed that the organism identified was Brucella, specifically with the sequence number 289 and a relative abundance of 90.881%. Consequently, the treatment for combating Brucella infection involved the administration of enteric-coated capsules containing doxycycline hydrochloride. The recommended dosage was one 100 mg capsule taken twice day, with an additional single capsule. Rifampicin capsules should be taken once a day. The recommended dosage is 1 capsule, containing 150 mg of the medication. In some cases, a higher initial dose of 4 capsules may be prescribed. An intravenous infusion of Dexamethasone 10 mg was administered daily. After a period of 12 days, the dosage was decreased to 30 mg/day of prednisone acetate tablets. Subsequently, the dosage was further reduced by 5 mg/day to reach a final dosage of 20 mg/day. Throughout the whole duration of the treatment, the patients received symptomatic interventions including dehydration to decrease intracranial pressure, electrolyte supplements, and stomach protection. The patient’s condition remained stable, with alleviation of clinical symptoms, and he adhered to medication for a duration of 3 months following discharge.

### Follow-up after discharge

The patient was reexamined in our hospital for three times at half a month, 1 month and 2 months after discharge (Table 1). The last reexamination result of CSF was: pressure 120mmH_2_O, white blood cells 21.00 × 10^6^/L, CSF protein 0.38 g/L, glucose 2.67 mmol/L and chlorine 125.8 mmol/L. At the same time, the serological tests of Brucella (Tiger red agglutination test and Tube agglutination test) were still negative at the second evaluation. Following discharge, the patient’s clinical symptoms and indicators resolved, and his mental state exhibited positive improvement.

**Table 1:** Results of CSF examinations of the patients at admission and follow-up after discharge.

Laboratory test indicators of cerebrospinal fluid	The first examination at admission	The second examination in the hospital	15 days after discharge	1 month after discharge	2 months after discharge	Reference range
Cerebrospinal fluid pressure mmHg	260	210	215	200	120	80–180
White blood cells*10^6^	597	111	54	20	21	0–8
Protein g/L	1.22	0.59	1.16	0.4	0.38	0.2–0.4
Glucose mmol/L	<1.0	<1.1	1.7	2.4	2.67	2.5–4.4
Chlorine mmol/L	119.0	118.0	119.1	123.4	125.8	120–130
Fungal and bacterial cultures	Negative	Negative	Negative	Negative	Negative	Negative

## Discussion

Brucella is a kind of Gram-negative bacilli, with a total of 6 species, among which 4 species of Brucella from cattle, sheep, pigs and dogs can cause human brucellosis (10). In the endemic areas of brucellosis, due to the close contact between animals and humans, the infection rate is relatively high (11). It has been reported that the estimated actual incidence of brucellosis is between 5 million and 12.5 million cases per year (12). Neurobrucellosis is a rare complication of brucellosis, accounting for approximately 2% to 10% of brucellosis, with serious clinical manifestations and complicated pathogenesis (2). Bacteria, toxins and allergic reactions all participate in the occurrence and development of the disease to varying degrees (13, 14). Understanding the pathogenesis of brucellosis encephalopathy will contribute to better diagnosis and treatment. Blood–brain barrier is a strong and highly adjustable physical barrier, which plays a good role in blocking exogenous neurotoxic components (15, 16). Therefore, limited by the blood–brain barrier, Brucella can not directly enter the CNS (15). Recent research has shown that Brucella is able to adhere to, invade, and reproduce within brain microvascular endothelial cells. However, the bacteria are unable to pass through the layer of these cells on their own. Instead, they are able to pass through the brain microvascular endothelial cells when they are carried by infected monocytes, allowing them to invade the CNS (17). Studies have also indicated that brute bacteria, as an invisible pathogen, has an inherent immunity that escapes and resists the destruction of swallowing cells (18, 19). And after bacteria invade the host, neutrophils are the first immune cells to encounter and devour Brucella, and the phagocytized Brucella can survive in neutrophils for a long time and induce neutrophils to die prematurely (20, 21). This is consistent with the fact that the number of neutrophils infected by Brucella in the target organ is small during Brucella infection, and some patients have neutropenia in peripheral blood (20, 21), and the white blood cells in our reported cases are also reduced by routine blood examination.

Neurobrucellosis has many forms, such as encephalitis, meningitis, meningoencephalitis, myelitis and increased intracranial pressure. It is characterized by headache, blindness, numbness of limbs, weakness and hyperalgesia (4). However, because some patients with systemic brucellosis without nervous system involvement and patients with infectious meningitis caused by other microorganisms may have similar symptoms, clinical diagnosis of neurobrucellosis requires direct or indirect evidence of brucellosis in CSF. However, the pathogenic microorganisms have a long culture period and low positive rate, which is easy to be misdiagnosed and delay treatment. mNGS technology is a new method in microbiological diagnosis, which can detect all microorganisms in samples at one time, including viruses, bacteria, fungi and parasites, and has important diagnostic value for infectious diseases of the CNS (22, 23). It has been reported that a study in the northwest region of China (endemic area of brucellosis for this case) found that from 2015 to 2021, the sensitivity of mNGS in detecting Brucella in cerebrospinal fluid was 90%, while the sensitivity of cerebrospinal fluid culture was 54.5% (24). Researchers detected 11~104 Brucella-specific sequences by metagenomic sequencing technology in CSF of 4 patients with nervous system diseases (22). In this case, the serological test (Tiger red agglutination test and Tube agglutination test) of Brucella was negative, and the next-generation sequencing of CSF metagenome suggested that the sequence number of Brucella was 289, and the relative abundance was 90.881%, thus the diagnosis was neurobrucellosis.

In addition, similar cases have been reported by other researchers. Haji-Abdolbagi et al. (25)reviewed the clinical symptoms of 31 individuals with neurobrucellosis. They discovered that 2 instances had negative results in the serum test Tube agglutination test, while 1 case had negative results in both the CSF and serological test Tube agglutination test, as well as in culture. Neurobrucellosis was diagnosed based only on the anomalous disappearance of CSF fluid and the clinical response observed after anti-brucellosis medication. Tekin-Koruk et al. (26)reported a case of neurobrucellosis, in which both serum and CSF were negative for brucellosis agglutination test, but brucellosis was cultured in CSF in automatic blood culture system. Papadopoulos et al. (27) reported a patient who suffered from systemic brucellosis 2 years before admission, and after successful treatment, the serological examination turned negative and the PCR examination of CSF was negative during the whole course, but the 16SrRNA sequencing of CSF showed the existence of Brucella. Qasim et al. (28) reported a case of brucellosis encephalopathy with acute ataxia and slurred speech. The test Tube agglutination test of CSF and neuroimaging examination were positive, but the test Tube agglutination test of brucellosis serology was negative. In addition, CSF samples were sent to specialized laboratories, and Brucella was detected by PCR, and the results were negative. However, the samples used for testing were obtained a few days after the patient took ceftriaxone, and the patient was diagnosed with brucellosis 2 years ago. In this case, the patient was diagnosed with neurobrucellosis through the detection of Brucella in cerebrospinal fluid by mNGS and achieved a good prognosis. However, the serological tests of peripheral blood before and after treatment were negative. This suggests that the difference between the serological test results of peripheral blood and those of cerebrospinal fluid may be related to local Brucella infection and the specific immune response of the CNS. The patient in this case presented initially with neurobrucellosis, which is different from the common pathway of intracranial infection secondary to peripheral brucellosis. The pathogen directly colonized the CNS and formed a focal infection lesion, rather than causing a systemic infection through hematogenous dissemination. This led to an extremely low pathogen load in the peripheral blood or the lack of continuous antigen stimulation, making it difficult to trigger a systemic humoral immune response of sufficient intensity. Secondly, it should be noted that the inherent limitations of serological detection methods cannot be ignored. During the window period in the early infection stage or in the state of low antibody titers during the chronic stage, false-negative test results may occur. In addition, the physiological barrier function of the blood–brain barrier leads to the separation of the immune microenvironments in the CNS and the peripheral region (29, 30). The local immune response in the CNS often fails to synchronize with the antibody levels in the peripheral blood. The immune response in the CNS is mainly cell-mediated immunity (such as the infiltration of macrophages and T cells), while the humoral immune response is relatively weak. Moreover, specific antibodies are mainly synthesized locally, which makes it difficult to detect them in the peripheral blood (31). To sum up, serological examination is uncertain in the diagnosis of neurobrucellosis, which may be related to the patient’s individual situation, treatment and local recurrence. Therefore, we think that when patients have symptoms of nervous system infection with unknown causes, and when serological and imaging tests are negative, neurobrucella should be considered as a differential diagnosis. mNGS can help determine if brucella is present in the patient’s CSF and provide further treatment to reduce permanent neurological dysfunction.

In addition, mNGS has demonstrated unique advantages in the detection of pathogenic microorganisms in the intracranial region (32). However, its clinical application still faces multiple technical bottlenecks and challenges in diagnosis and treatment (33). Firstly, there is the problem of false-negative results in mNGS. The reasons involve multiple aspects such as sampling (insufficient pathogen load), storage and transportation (nucleic acid degradation due to the lack of low-temperature conditions), experiments, the database (new pathogens not covered), and methodology (a high proportion of human-derived sequences) (34). Secondly, the false-positive results of mNGS mislead or interfere with clinical diagnosis. The detection of microorganisms in clinical specimens by mNGS can reflect the normal microbiota, transiently colonized bacteria, sample contamination, or infection. Although cerebrospinal fluid is a commonly considered sterile sample, the possibility of contamination still cannot be ruled out. Therefore, further research is needed to find the best method for distinguishing between colonization and infection (35). In addition, mNGS has powerful diagnostic ability for the detection of new microorganisms. However, the discovery of a new pathogen or a group of unusual microorganisms in clinical samples only indicates its potential pathogenic role and cannot determine the causal relationship between it and the disease (36). Therefore, during the clinical diagnosis and treatment process, regarding the problem of false negatives, the sampling process should be optimized (such as increasing the sampling volume and selecting samples from the infection foci), the conditions for low-temperature transportation and preservation of samples should be strictly standardized, the experimental methods should be improved, the pathogen database should be updated regularly, and the host DNA removal technology should be adopted to reduce the interference of human-derived sequences. Regarding the problem of false positives, it is necessary to establish a contamination control system, verify the results by combining clinical indicators with traditional detection methods (culture, PCR), and at the same time, develop a scoring system based on machine learning to distinguish between colonization and infection. In terms of the identification of new pathogens, a multi-center collaboration mechanism should be established. Their pathogenicity should be verified through epidemiological investigations, animal models, and molecular biology experiments, and the results should be dynamically correlated with the patients’ clinical manifestations and treatment effects for comprehensive analysis.

## Data Availability

The original contributions presented in the study are included in the article/supplementary material, further inquiries can be directed to the corresponding author.
